# Maximum Causal Entropy Specification Inference from Demonstrations

**DOI:** 10.1007/978-3-030-53291-8_15

**Published:** 2020-06-16

**Authors:** Marcell Vazquez-Chanlatte, Sanjit A. Seshia

**Affiliations:** 8 Microsoft Research Lab, Redmond, WA USA; 9 University of Southern California, Los Angeles, CA USA; University of California, Berkeley, USA

## Abstract

In many settings, such as robotics, demonstrations provide a natural way to specify tasks. However, most methods for learning from demonstrations either do not provide guarantees that the learned artifacts can be safely composed or do not explicitly capture temporal properties. Motivated by this deficit, recent works have proposed learning Boolean *task specifications*, a class of Boolean non-Markovian rewards which admit well-defined composition and explicitly handle historical dependencies. This work continues this line of research by adapting maximum *causal* entropy inverse reinforcement learning to estimate the posteriori probability of a specification given a multi-set of demonstrations. The key algorithmic insight is to leverage the extensive literature and tooling on reduced ordered binary decision diagrams to efficiently encode a time unrolled Markov Decision Process. This enables transforming a naïve algorithm with running time exponential in the episode length, into a polynomial time algorithm.

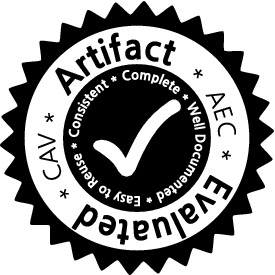



## Introduction

**Fig. 1.**
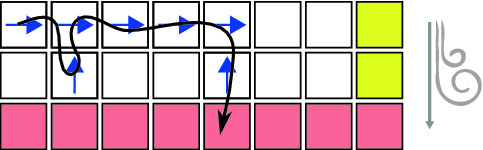
Example of an agent unsuccessfully demonstrating the task “reach a yellow tile while avoiding red tiles”. (Color figure online)

In many settings, episodic demonstrations provide a natural and robust mechanism to partially specify a task, even in the presence of errors. For example, consider the agent operating in the gridworld illustrated in Fig. 1. Blue arrows denote intended actions and the solid black arrow shows the agent’s actual path. This path can stochastically differ from the blue arrows due to a downward wind. One might naturally ask: “What task was this agent attempting to perform?” Even without knowing if this was a positive or negative example, based on the agent’s state/action sequence, one can reasonably infer the agent’s intent, namely, “reach the yellow tile while avoiding the red tiles.” Compared with traditional learning from positive and negative examples, this is somewhat surprising, particularly given that the task is never actually demonstrated in Fig. 1.

This problem, inferring intent from demonstrations, has received a fair amount of attention over the past two decades particularly within the robotics community [5, 22, 30, 33]. In this literature, one traditionally models the demonstrator as operating within a dynamical system whose transition relation only depends on the current state and action (called the Markov condition). However, even if the dynamics are Markovian, many tasks are naturally modeled in history dependent (non-Markovian) terms, e.g., “if the robot enters a blue tile, then it must touch a brown tile *before* touching a yellow tile”. Unfortunately, most methods for learning from demonstrations either do not provide guarantees that the learned artifacts (e.g. rewards) can be safely composed or do not explicitly capture history dependencies [30].

Motivated by this deficit, recent works have proposed specializing to **task specifications**, a class of Boolean non-Markovian rewards induced by formal languages. This additional structure admits well-defined compositions and explicitly captures temporal dependencies [15, 30]. A particularly promising direction has been to adapt maximum entropy inverse reinforcement learning [33] to task specifications, enabling a form of robust specification inference, even in the presence unlabeled demonstration errors [30].

However, while powerful, the principle of maximum entropy is limited to settings where the dynamics are deterministic or agents that use open-loop policies [33]. This is because the principle of maximum entropy incorrectly allows the agent’s predicted policy to depend on future state values resulting in an overly optimistic agent [19]. For instance, in our gridworld example (Fig. 1), the principle of maximum entropy would discount the possibility of slipping, and thus we would not forecast the agent to correct its trajectory after slipping once.

This work continues this line of research by instead using the principle of maximum *causal* entropy, which generalizes the principle of maximum entropy to general stochastic decision processes [32]. While a conceptually straightforward extension, a naïve application of maximum *causal* entropy inverse reinforcement learning to non-Markovian rewards results in an algorithm with run-time exponential in the episode length, a phenomenon sometimes known as the **curse of history** [24]. The key algorithmic insight in this paper is to leverage the extensive literature and tooling on Reduced Ordered Binary Decision Diagrams (BDDs) [3] to efficiently encode the time unrolled composition of the dynamics and task specification. This allows us to translate a naïve exponential time algorithm into a polynomial time algorithm. In particular, we shall show that this BDD has size at most linear in the episode length making inference comparatively efficient.

### Related Work

Our work is intimately related to the fields of Inverse Reinforcement Learning and Grammatical Inference. **Grammatical inference** [8] refers to the well-developed literature on learning a formal grammar (often an automaton) from data. Examples include learning the smallest automata that in consistent with a set of positive and negative strings [7, 8] or learning an automaton using membership and equivalence queries [1]. This and related work can be seen as extending these methods to unlabeled and potentially noisy demonstrations, where demonstrations differ from examples due to the existence of a dynamics model. This notion of demonstration derives from the Inverse Reinforcement Learning literature.

In **Inverse Reinforcement Learning** (IRL) [22] the demonstrator, operating in a stochastic environment, is assumed to attempt to (approximately) optimize some unknown reward function over the trajectories. In particular, one traditionally assumes a trajectory’s reward is the sum of state rewards of the trajectory. This formalism offers a succinct mechanism to encode and generalize the goals of the demonstrator to new and unseen environments.

In the IRL framework, the problem of learning from demonstrations can then be cast as a Bayesian inference problem [25] to predict the most probable reward function. To make this inference procedure well-defined and robust to demonstration/modeling noise, Maximum Entropy [33] and Maximum Causal Entropy [32] IRL appeal to the principles of maximum entropy [13] and maximum causal entropy respectively [32]. This results in a likelihood over the demonstrations which is no more committed to any particular behavior than what is required to match observed statistical features, e.g., average distance to an obstacle. While this approach was initially limited to rewards represented as linear combinations of scalar features, IRL has been successfully adapted to arbitrary function approximators such as Gaussian processes [20] and neural networks [5]. As stated in the introduction, while powerful, traditional IRL provides no principled mechanism for composing the resulting rewards.

**Compositional RL:** To address this deficit, composition using soft optimality has recently received a fair amount of attention; however, the compositions are limited to either strict disjunction (do X *or* Y) [26, 27] or conjunction (do X *and* Y) [6]. Further, this soft optimality only bounds the deviation from simultaneously optimizing both rewards. Thus, optimizing the composition does not preclude violating safety constraints embedded in the rewards (e.g., do not enter the red tiles).

**Logic Based IRL:** Another promising approach for introducing compositionality has been the recent research on automata and logic based encodings of rewards [11, 14] which admit well defined compositions. To this end, work has been done on inferring Linear Temporal Logic (LTL) formulas by finding the specification that minimizes the expected number of violations by an optimal agent compared to the expected number of violations by an agent applying actions uniformly at random [15]. The computation of the optimal agent’s expected violations is done via dynamic programming on the explicit product of the deterministic Rabin automaton [4] of the specification and the state dynamics. A fundamental drawback of this procedure is that due to the curse of history, it incurs a heavy run-time cost, even on simple two state and two action Markov Decision Processes. Additionally, as with early work on grammatical inference and IRL, these techniques do not produce likelihood estimates amenable to Bayesian inference.

**Maximum Entropy Specification Inference:** In our previous work [30], we adapted maximum entropy IRL to learn task specifications. Similar to standard maximum entropy IRL, this technique produces robust likelihood estimates. However, due to the use of the principle of maximum entropy, rather than maximum *causal* entropy, this model is limited to settings where the dynamics are deterministic or agents with open-loop policies [33].

**Inference Using BDDs:** This work makes heavy use of Binary Decision Diagrams (BDDs) [3] which are frequently used in symbolic value iteration for Markov Decision Processes [9] and reachability analysis for probabilistic systems [18]. However, the literature has largely relied on Multi-Terminal BDDs to encode the transition probabilities for a **single** time step. In contrast, this work introduces a two-terminal encoding based on the finite unrolling of a probabilistic circuit. To the best of our knowledge, the most similar usage of BDDs for inference appears in the independently discovered literal weight based encoding of [10] - although their encoding does not directly support non-determinism or state-indexed random variables.

**Contributions:** The primary contributions of this work are two fold. First, we leverage the principle of maximum causal entropy to provide the likelihood of a specification given a set of demonstrations. This formulation removes the deterministic and/or open-loop restriction imposed by prior work based on the principle of maximum entropy. Second, to mitigate the curse of history, we propose using a BDD to encode the time unrolled Markov Decision Process that the maximum causal entropy forecaster is defined over. We prove that this BDD has size that grows linearly with the horizon and quasi-linearly with the number of actions. Furthermore, we prove that our derived likelihood estimates are robust to the particular reward associated with satisfying the specification. Finally, we provide an initial experimental validation of our method. An overview of this pipeline is provided in Fig. 8.

## Problem Setup

We seek to learn task specifications from demonstrations provided by a teacher who executes a sequence of actions that probabilistically change the system state. For simplicity, we assume that the set of actions and states are finite and fully observed. Further, until Sect. 5.3, we shall assume that all demonstrations are a fixed length, $\tau \in \mathbb {N}$. Formally, we begin by modeling the underlying dynamics as a probabilistic automaton.
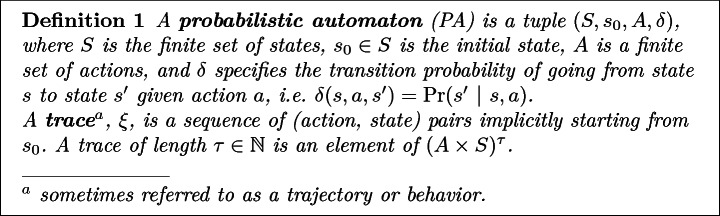


**Fig. 2.**
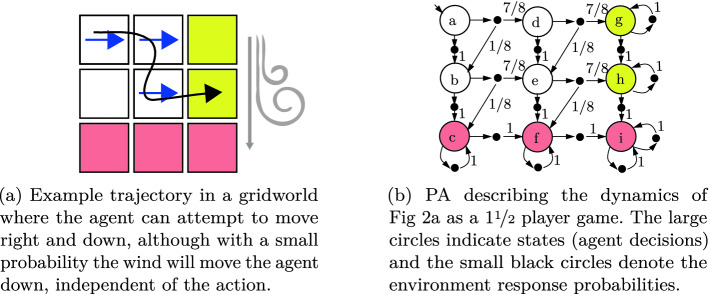
Example of gridworld probabilistic automata (PA).


Note that probabilistic automata are equivalently characterized as $1\nicefrac {1}{2} player games$ where each round has the agent choose an action and then the environment samples a state transition outcome. In fact, this alternative characterization is implicitly encoded in the directed bipartite graph used to visualize probabilistic automata (see Fig. 2b). In this language, we refer to the nodes where the agent makes a decision as a **decision node** and the nodes where the environment samples an outcome as a **chance node**.

Next, we develop machinery to distinguish between desirable and undesirable traces. For simplicity, we focus on finite trace properties, referred to as specifications, that are decidable within some fixed $\tau \in \mathbb {N}$ time steps, e.g., “Recharge before t = 20.”

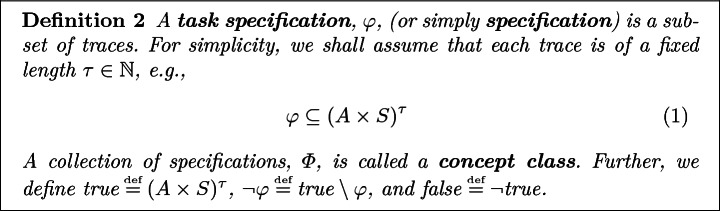


Often specifications are not directly given as sets, but induced by abstract descriptions of a task. For example, the task “avoid lava” induces a concrete set of traces that never enter lava tiles. If the workspace/world/dynamics change, this abstract specification would map to a different set of traces.

### Specification Inference from Demonstrations

The primary task in this paper is to find the specification that best explains/forecasts the behavior of an agent. As in our prior work [30], we formalize our problem statement as:

**Figure:**
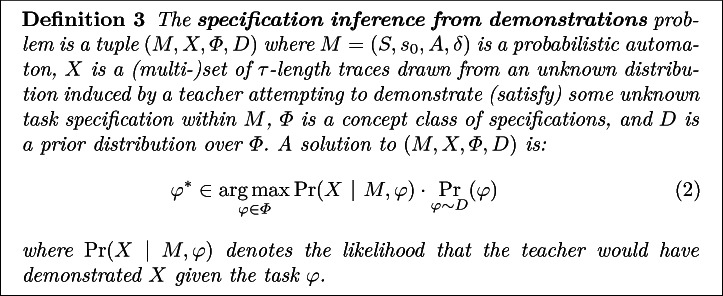

Of course, by itself, the above formulation is ill-posed as $\Pr (X~|~M, \varphi )$ is left undefined. Below, we shall propose leveraging Maximum Causal Entropy Inverse Reinforcement Learning (IRL) to select the demonstration likelihood distribution in a regret minimizing manner.

## Leveraging Inverse Reinforcement Learning

The key idea of Inverse Reinforcement Learning (IRL), or perhaps more accurately Inverse Optimal Control, is to find the reward structure that best explains the actions of a reward optimizing agent operating in a Markov Decision Process. We formalize below. 
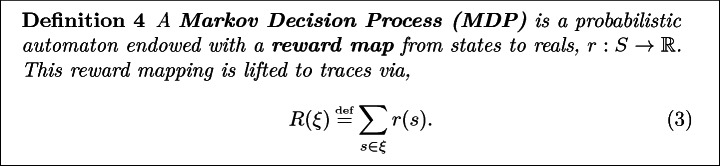


### Remark 1

Note that a temporal discount factor, $\gamma \in [0, 1]$ can be added into (3) by introducing a sink state, $\$$, to the MDP, where $r(\$) = 0$ and

4 $$\begin{aligned} \Pr (s' = \$~|~s, a) = {\left\{ \begin{array}{ll} \gamma &{} \text {if } s \ne \$\\ 1 &{} \text {otherwise} \end{array}\right. }. \end{aligned}$$

Given a MDP, the goal of an agent is to maximize the expected trace reward. In this work, we shall restrict ourselves to rewards that are given as a linear combination of **state features**, $\mathbf {f}: S \rightarrow \mathbb {R}_{\ge 0}^n$, e.g.,

5 $$\begin{aligned} r(s) = \mathbf {\theta } \cdot \mathbf {f}(s) \end{aligned}$$

for some $\mathbf {\theta } \in \mathbb {R}^n$. Note that since state features can themselves be rewards, such a restriction does not actually restrict the space of possible rewards.

### Example 1

Let the components of $\mathbf {f}(s)$ be distances to various locations on a map. Then the choice of $\mathbf {\theta }$ characterizes the relative preferences in avoiding/reaching the respective locations.

Formally, we model an agent as acting according to a **policy**.
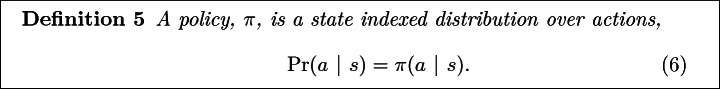


In this language, the agent’s goal is equivalent to finding a policy which maximizes the expected trace reward. We shall refer to a trace generated by such an agent as a **demonstration**. Due to the Markov requirement, the likelihood of a demonstration, $\xi$, given a particular policy, $\pi$, and probabilistic automaton, *M*, is easily stated as:

7 $$\begin{aligned} \Pr (\xi ~|~M, \pi ) = \prod _{s', a, s \in \xi }\Pr (s'~|~s, a)\cdot \Pr (a~|~s). \end{aligned}$$

Thus, the likelihood of multi-set of i.i.d demonstrations, *X*, is given by:

8 $$\begin{aligned} \Pr (X~|~M, \pi ) = \prod _{\xi \in X}\Pr (\xi ~|~M, \pi ). \end{aligned}$$

### Inverse Reinforcement Learning (IRL)

As previously stated, the main motivation in introducing the MDP formalism has been to discuss the inverse problem. Namely, given a set of demonstrations, find the reward that best “explains” the agent’s behavior, where by “explain” one typically means that under the conjectured reward, the agent’s behavior was approximately optimal. Notice however, that many undesirable rewards satisfy this property. For example, consider the following reward in which every demonstration is optimal,

9 $$\begin{aligned} r: s \mapsto 0. \end{aligned}$$

Furthermore, observe that given a fixed reward, many policies are approximately optimal! For instance, using (9), an optimal agent could pick actions uniformly at random or select a single action to always apply.

### Maximum Causal Entropy IRL

A popular, and in practice effective, solution to the lack of unique policy conundrum is to appeal to the **principle of maximum causal entropy** [32]. To formalize this principle, we recall the definitions of causally conditioned probability [17] and causal entropy [17, 23].

**Figure:**
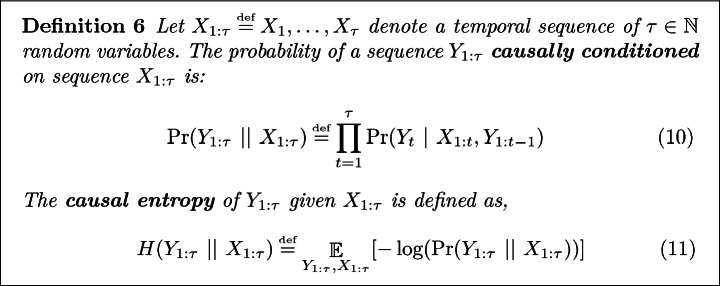

In the case of inverse reinforcement learning, the principle of maximum causal entropy suggests forecasting using the policy whose action sequence, $A_{1:\tau }$, has the highest causal entropy, conditioned on the state sequence, $S_{1:\tau }$. That is, find the policy that maximizes

12 $$\begin{aligned} H(A_{1:\tau }~||~S_{1:\tau }), \end{aligned}$$

subject to feature matching constraints, $\mathop {\mathbb {E}}[\mathbf {f}]$, e.g., does the resulting policy, $\pi ^*$, complete the task as seen in the data. Compared to all other policies, this policy (i) minimizes regret with respect to model/reward uncertainty, (ii) ensures that the agent’s predicted policy does not depend on the future, (iii) is consistent with observed feature statistics [32].

Concretely, as proved in [32], when an agent is attempting to maximize the sum of feature state rewards, $\sum _{t=1}^T\mathbf {\theta } \cdot \mathbf {f}(s_t)$, the principle of maximum causal entropy prescribes the following policy: 
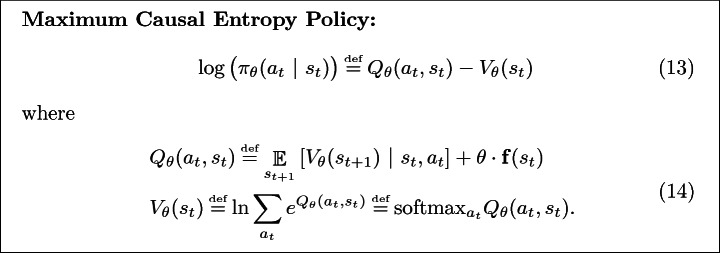


where, $\theta$ is such that (14) results in a policy which matches feature demonstrations.

#### Remark 2

Note that replacing softmax with max in (14) yields the standard Bellman Backup [2] used to compute the optimal policy in tabular reinforcement learning. Further, it can be shown that maximizing causal entropy corresponds to believing that the agent is exponentially biased towards high reward policies [32]:

15 $$\begin{aligned} \Pr (\pi _\theta ~|~M) \propto \exp \Big (\mathop {\mathbb {E}}_{\begin{array}{c} \xi \end{array}}[R_\theta (\xi )~|~\pi _\theta , M]\Big ), \end{aligned}$$

where (14) is the most likely policy under (15).

#### Remark 3

In the special case of scalar state features, $\mathbf {f}: S \rightarrow \mathbb {R}_{\ge 0}$, the maximum causal entropy policy (14) becomes increasingly optimal as $\theta \in \mathbb {R}$ increases (since softmax monotonically approaches max). In this setting, we shall refer to $\theta$ as the agent’s **rationality coefficient**.

### Non-Markovian Rewards

The MDP formalism traditionally requires that the reward map be Markovian (i.e., state based); however, in practice, many tasks are history dependent, e.g. touch a red tile and then a blue tile.

A common trick within the reinforcement learning literature is to simply change the MDP and add the necessary history to the state so that the reward is Markovian, e.g. a flag for touching a red tile. However, in the case of inverse reinforcement learning, by definition, one does not know what the reward is. Therefore, one cannot assume to a priori know what history suffices.

Further exacerbating the situation is the fact that naïvely including the entire history into the state results in an exponential increase in the number of states. Nevertheless, as we shall soon see, by restricting the class of rewards to represent task specifications, this curse can be mitigated to only result in a blow-up that is at most **linear** in the state space size and in the trace length!

To this end, we shall find it fruitful to develop machinery for embedding the full trace history into the state space. Explicitly, we shall refer to the process of adding all history to a probabilistic automaton’s (or MDP’s) state as **unrolling**.

**Figure:**
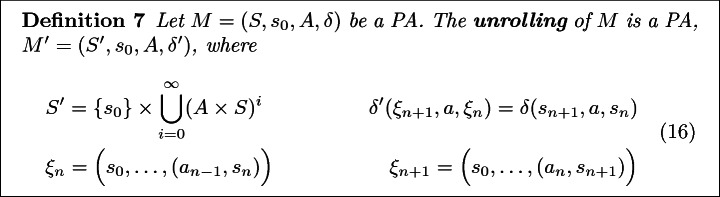

If $R : S^\tau \rightarrow \mathbb {R}$ is a non-Markovian reward over $\tau$-length traces, then we endow the corresponding unrolled PA with the now Markovian Reward,17Further, by construction the reward is Markovian in $S'$ and only depends only $\tau$-length state sequences,

18 $$\begin{aligned} \sum _{t=0}^\infty r'\big ((s_0, a_0), \ldots s_\tau \big ) = R(s_0, \ldots , s_\tau ). \end{aligned}$$

**Fig. 3.**
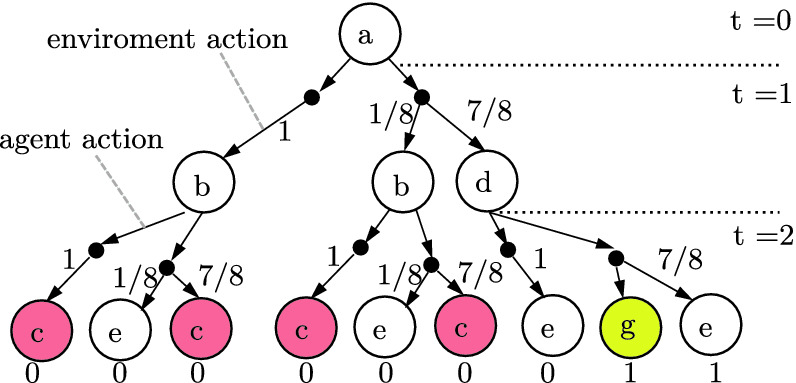
Decision tree generated by the PA shown in Fig. 2 and specification “By $\tau =2$, reach a yellow tile while avoiding red tiles.”. Here a binary reward is given depending on whether or not the agent satisfies the specification. (Color figure online)

Next, observe that for $\tau$-length traces, the 1$\nicefrac {1}{2}$ player game formulation’s bipartite graph forms a tree of depth $\tau$ (see Fig. 3). Further, observe that each leaf corresponds to unique $\tau$-length trace. Thus, to each leaf, we associate the corresponding trace’s reward, $R(\xi )$. We shall refer to this tree as a **decision tree**, denoted $\mathbb {T}$.

Finally, observe that the trace reward depends only on the sequence of agent actions, *A*, and environment actions, $A_e$. That is, $\mathbb {T}$ can be interpreted as a function:

19 $$\begin{aligned} \mathbb {T} : {(A \times A_e)^\tau } \rightarrow \mathbb {R}. \end{aligned}$$

### Specifications as Non-Markovian Rewards

Next, with the intent to frame our specification inference problem as an inverse reinforcement learning problem, we shall overload notation and denote by $\varphi$ the following non-Markovian reward corresponding to a specification $\varphi \in (A \times S)^\tau$,20Note that the corresponding decision tree is then a Boolean predicate:

21 $$\begin{aligned} \mathbb {T}_\varphi : {(A \times A_e)^\tau } \rightarrow \{0, 1\}. \end{aligned}$$

### Computing Maximum Causal Entropy Specification Policies

Now let us return to the problem of computing the policy prescribed by (14). In particular, note that viewing the unrolled reward (17) as a scalar state feature results in the following soft-Bellman Backup:

22 $$\begin{aligned} \begin{aligned}&Q_{\mathbf {\theta }}(a_t, \xi _t) = \mathop {\mathbb {E}}\left[ V_{\mathbf {\theta }}(s_{t+1})~|~\xi _t, a_t\right] \\&V_{\mathbf {\theta }}(\xi _t) = {\left\{ \begin{array}{ll} \theta \cdot \varphi (\xi _t) &{} \text {if } t = \tau \\ \mathrm {softmax}_{a_t} Q_{\mathbf {\theta }}(a_t, \xi _t) &{} \text {otherwise} \end{array}\right. } \end{aligned}, \end{aligned}$$

where $\xi _i \in \{s_0\}\times (A\times S)^i$ denotes a state in the unrolled MDP.

Equation (22) thus suggests a naïve dynamic programming scheme over $\mathbb {T}$ starting at the $t=\tau$ leaves to compute $Q_\theta$ and $V_\theta$ (and thus $\pi _{\mathbf {\theta }}$).

**Fig. 4.**
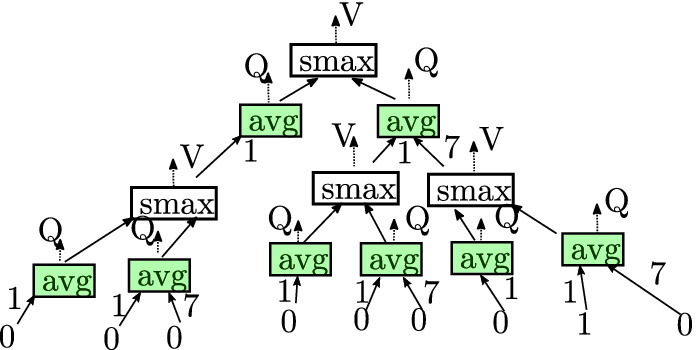
Computation graph generated from applying (14) to the decision tree shown in Fig. 3. Here smax and avg denote the softmax and weighted average respectively.

Namely, in $\mathbb {T}$, the chance nodes, which correspond to action/state pairs, are responsible for computing *Q* values and the decision nodes, which correspond to states waiting for an action to be applied, are responsible for computing *V* values. For chance nodes this is done by taking the $\mathrm {softmax}$ of the values of the child nodes. Similarly, for decision nodes, this is done by taking a weighted average of the child nodes, where the weights correspond to the probability of a given transition. This, at least conceptually, corresponds to transforming $\mathbb {T}$ into a bipartite computation graph (see Fig. 4).

Next, note that (i) the above dynamic programming scheme can be trivially modified to compute the expected trace reward of the maximum causal entropy policy and (ii) the expected reward increases^1^ with the rationality coefficient $\theta$.

Observe then that, due to monotonicity, bisection (binary search) approximates $\theta$ to tolerance $\epsilon$ in $O(\log (1/\epsilon ))$ time. Additionally, notice that the likelihood of each demonstration can be computed by traversing the path of length $\tau$ in $\mathbb {T}$ corresponding to the trace and multiplying the corresponding policy and transition probabilities (8). Therefore, if $|A_e| \in \mathbb {N}$ denotes the maximum number of outcomes the environment can choose from (i.e, the branching factor for chance nodes), it follows that the run-time of this naïve scheme is:

23 $$\begin{aligned} O\bigg (\overbrace{\underbrace{\Big (|A|\cdot |A_e|\Big )^\tau }_{|\mathbb {T}|} ~\cdot \underbrace{\log (1/\epsilon )}_{\text {Feature Matching}}}^{\text {compute policy}} + \underbrace{\tau |X|}_{\text {evaluate demos}}\bigg ). \end{aligned}$$

### Task Specification Rewards

Of course, the problem with this naïve approach is that explicitly encoding the unrolled tree, $\mathbb {T}$, results in an exponential blow-up in the space and time complexity. The key insight in this paper is that the additional structure of task specifications enables avoiding such costs while still being expressive. In particular, as is exemplified in Fig. 4, the computation graphs for task specifications are often highly redundant and apt for compression.

**Fig. 5.**
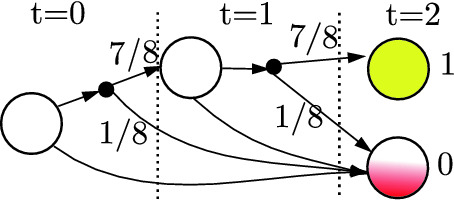
Reduction of the decision tree shown in Fig. 3.

In particular, we shall apply the following two semantic preserving transformations: (i) Eliminate nodes whose children are isomorphic sub-graphs, i.e., inconsequential decisions (ii) Combine all isomorphic sub-graphs i.e., equivalent decisions. We refer to the limit of applying these two operations as a **reduced ordered probabilistic decision diagram** and shall denote^2^ the reduced variant of $\mathbb {T}$ as $\mathcal {T}$.

#### Remark 4

For those familiar, we emphasize that these decision diagrams are MDPs, not Binary Decision Diagrams (see Sect. 4). Importantly, more than two actions can be taken from a node if $\max (|A|, |A_e|) \ge 2$ and $A_e$ has a state dependent probability distribution attached to it. That said, the above transformations are **exactly** the reduction rules for BDDs [3].

As Fig. 5 illustrates, reduced decision diagrams can be much smaller than their corresponding decision tree. Nevertheless, we shall briefly postpone characterizing $|\mathcal {T}|$ until developing some additional machinery in Sect. 4. Computationally, three problems remain.

1. How can our naïve dynamic programming scheme be adapted to this compressed structure. In particular, because many interior nodes have been eliminated, one must take care when applying (22).
2. How do concrete demonstrations map to paths in the compressed structure when evaluating likelihoods (8).
3. How can one construct $\mathcal {T}$ without first constructing $\mathbb {T}$, since failing to do so would negate any complexity savings.

We shall postpone discussing solutions to the second and third problems until Sect. 4. The first problem however, can readily be addressed with the tools at hand. Recall that in the variable ordering, nodes alternate between decision and chance nodes (i.e., agent and environment decisions), and thus alternate between taking a softmax and expectations of child values in (22). Next, by definition, if a node is skipped in $\mathcal {T}$, then it must have been inconsequential. Thus the trace reward must have been independent of the decision made at that node. Therefore, the softmax/expectation’s corresponding to eliminated nodes must have been over a constant value - otherwise the eliminated sequences would be distinguishable w.r.t $\varphi$. The result is summarized in the following identities, where $\alpha$ denotes the value of an eliminated node’s children.

24 $$\begin{aligned} \mathrm {softmax}(\overbrace{\alpha , \ldots , \alpha }^{|A|}) = \log (e^\alpha + \ldots + e^\alpha ) = \ln (|A|) + \alpha \end{aligned}$$

25 $$\begin{aligned} \mathop {\mathbb {E}}_x[\alpha ] = \sum _{x}p(x)\alpha = \alpha \end{aligned}$$

Of course, it could also be the case that a sequence of nodes is skipped in $\mathcal {T}$. Using (24), one can compute the change in value, $\varDelta$, that the eliminated sequence of *n* decision nodes and any number of chance nodes would have applied in $\mathbb {T}$:

26 $$\begin{aligned} \varDelta (n, \alpha ) = \ln (|A|^n) + \alpha = n \ln (|A|) + \alpha \end{aligned}$$

Crucially, evaluation of this compressed computation graph is linear in $|\mathcal {T}|$ which as shall later prove, is often much smaller than $|\mathbb {T}|$.

## Constructing and Characterizing $\mathcal {T}$

Let us now consider how to avoid the construction of $\mathbb {T}$ and characterize the size of the reduced ordered decision diagram, $\mathcal {T}$. We begin by assuming that the underlying dynamics is well-approximated in the random-bit model.
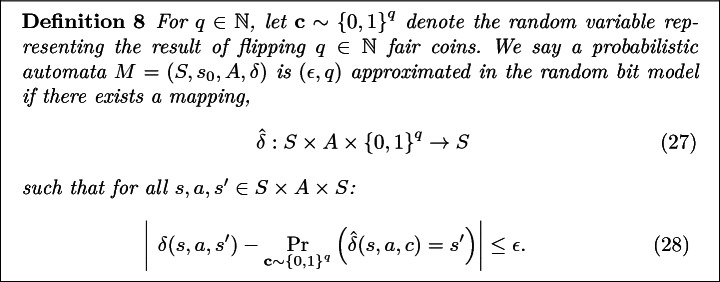


For example, in our gridworld example (Fig. 2a), if $\mathbf {c} \in \left\{ 0, 1\right\} ^3$, elements of *s* are interpreted as pairs in $\mathbb {R}^2$, and the right/down actions are interpreted as the addition of the unit vectors (1, 0) and (0, 1) then,

29 $$\begin{aligned} \hat{\delta }(s, a, \mathbf {c}) = {\left\{ \begin{array}{ll} s &{} \text {if } \max \nolimits _i[(s + a)_i] > 1\\ s + (0, 1) &{} \text {else if } \mathbf {c} = 0\\ s + a &{} \text {otherwise} \end{array}\right. }, \end{aligned}$$

As can be easily confirmed, (29) satisfies (28) with $\epsilon = 0$. In the sequel, we shall take access to $\hat{\delta }$ as given^3^. Further, to simplify exposition, until Sect. 5.1, we shall additionally require that the number of actions, |*A*|, be a power of 2. This assumption implies that *A* can be encoded using exactly $\log _2(|A|)$ bits.

Under the above two assumptions, the key observation is to recognize that $\mathbb {T}$ (and thus $\mathcal {T}$) can be viewed as a Boolean predicate over an alternating sequence of action bit strings and coin flip outcomes determining if the task specification is satisfied, i.e.,

30 $$\begin{aligned} \mathbb {T} : \{0, 1\}^{n} \rightarrow \{0, 1\}, \end{aligned}$$

where . That is to say, the resulting decision diagram can be re-encoded as a reduced ordered **binary** decision diagram [3].

**Figure:**
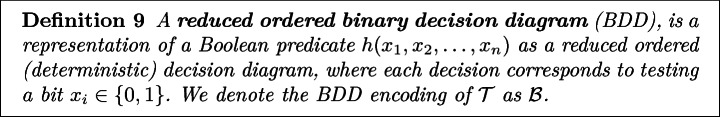

Binary decision diagrams are well developed both in a theoretical and practical sense. Before exploring these benefits, we first note that this change has introduced an additional problem. First, note that in $\mathcal {B}$, decision and chance nodes from $\mathbb {T}$ are now encoded as sequences of decision and chance nodes. For example, if $a \in A$ is encoded by the 4-length bit sequence $b_1b_2b_3b_4$, then four decisions are made by the agent before selecting an action. Notice however that the original semantics are preserved due to associativity of the $\mathrm {softmax}$ and $\mathop {\mathbb {E}}$ operators. In particular, recall that by definition,31and thus the semantics of the sequence decision nodes is equivalent to the decision node in $\mathbb {T}$. Similarly, recall that the coin flips are fair, and thus expectations are computed via $\text {avg}(\alpha _1, \ldots , \alpha _n) = \nicefrac {1}{n}(\sum _{i=1}^n \alpha _i)$. Therefore, averaging over two sequential coin flips yields,32which by assumption (28), is the same as applying $\mathop {\mathbb {E}}$ on the original chance node. Finally, note that skipping over decisions needs to be adjusted slightly to account for sequences of decisions. Recall that via (26), the corresponding change in value, $\varDelta$, is a function of initial value, $\alpha$, and the number of agent actions skipped, i.e., $|A|^n$ for *n* skipped decision nodes. Thus, in the BDD, since each decision node has two actions, skipping *k* decision bits corresponds to skipping $2^k$ actions. Thus, if *k* decision bits are skipped over in the BDD, the change in value, $\varDelta$, becomes,

33 $$\begin{aligned} \varDelta (k, \alpha ) = \alpha + k\ln (2). \end{aligned}$$

Further, note that $\varDelta$ can be computed in constant time while traversing the BDD. Thus, the dynamic programming scheme is linear in the size of $\mathcal {B}$.

### Size of $\mathcal {B}$

**Fig. 6.**
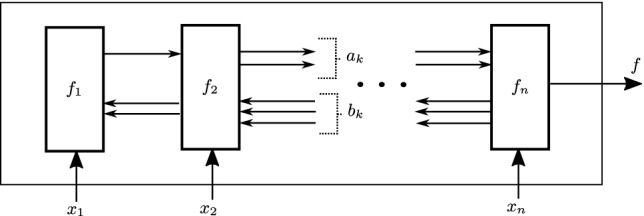
Generic network of Boolean modules for which Theorem 1 holds.

Next we return to the question of how big the compressed decision diagram can actually be. To this aim, we cite the following (conservative) bound on the size of an BDD given an encoding of the corresponding Boolean predicate in the linear model computation illustrated in Fig. 6 (for more details, we refer the reader to [16]).

In particular, consider an arbitrary Boolean predicate

34 $$\begin{aligned} f: \left\{ 0, 1\right\} ^{n} \rightarrow \left\{ 0, 1\right\} ^{} \end{aligned}$$

and a sequential arrangement of *n* Boolean modules, $f_1, f_2, \ldots , f_n$ where each $f_i$ has shape:

35 $$\begin{aligned} f_i : \left\{ 0, 1\right\} ^{} \times \left\{ 0, 1\right\} ^{a_{i-1}} \times \left\{ 0, 1\right\} ^{b_i} \rightarrow \left\{ 0, 1\right\} ^{a_i} \times \left\{ 0, 1\right\} ^{b_{i-1}}, \end{aligned}$$

and takes as input $x_i$ as well as $a_{i-1}$ outputs of its left neighbor and $b_i$ outputs of the right neighbor ($b_0 = 0, a_n = 1$). Further, assume that this arrangement is well defined, e.g. for each assignment to $x_1, \ldots , x_n$ there exists a unique way to set each of the inter-module wires. We say these modules compute *f* if the final output is equal to $f(x_1, \ldots , x_n)$.
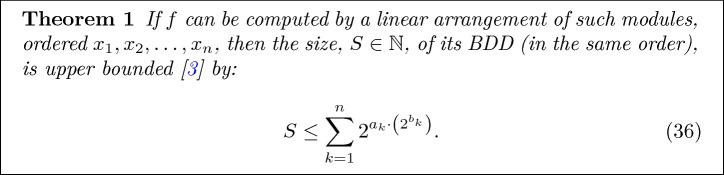


To apply this bound to our problem, recall that $\mathcal {B}$ computes a Boolean function where the decisions are temporally ordered and alternate between sequences of agent and environment decisions. Next, observe that because the traces are bounded (and all finite sets are regular), there exists a finite state machine which can monitor the satisfaction of the specification.

#### Remark 5

In the worst case, the monitor could be the unrolled decision tree, $\mathbb {T}$. This monitor would have exponential number of states. In practice, the composition of the dynamics and the monitor is expected to be much smaller.

**Fig. 7.**
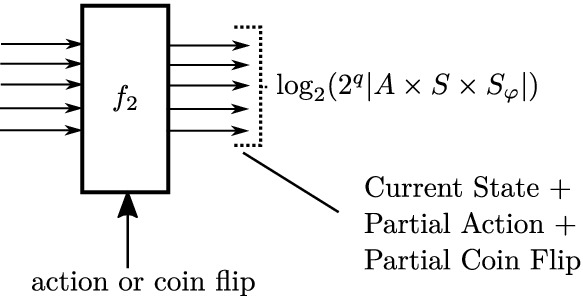
Generic module in linear model of computation for $\mathcal {B}$. Note that backward edges are not required.

Further, note that because this composed system is causal, no backward wires are needed, e.g., $\forall k~.~b_k = 0$. In particular, observe that because the composition of the dynamics and the monitor is Markovian, the entire system can be uniquely described using the monitor/dynamics state and agent/environment action (see Fig. 7). This description can be encoded in $\log _2(2^q|A \times S \times S_\varphi |)$ bits, where *q* denotes the number of coin flips tossed by the environment and $S_\varphi$ denotes the monitor state. Therefore, $a_k$ is upper bounded by $\log _2(2^q |A\,\times \,S\,\times \,S_\varphi |)$. Combined with (36) this results in the following bound on the size of $\mathcal {B}$.
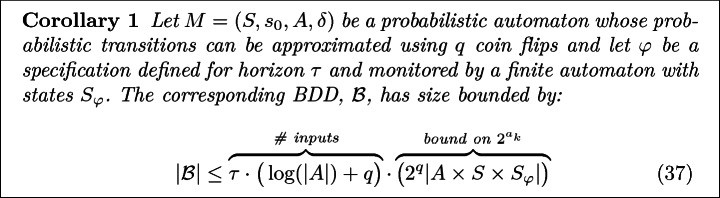

Notice that the above argument implies that as the episode length grows, $|\mathcal {B}|$ grows linearly in the horizon/states and quasi-linearly in the agent/environment actions!

#### Remark 6

Note that this bound actually holds for the minimal representation of the composed dynamics/monitor (even if it’s unknown a-prori!). For example, if the property is $true$, the BDD requires only one state (always evaluate true). This also illustrates that the above bound is often very conservative. In particular, note that for $\varphi = true$, $|\mathcal {B}| = 1$, independent of the horizon or dynamics. However, the above bound will always be linear in $\tau$. In general, the size of the BDD will depend on the particular symmetries compressed.

#### Remark 7

With hindsight, Corollary 1 is not too surprising. In particular, if the monitor is known, then one could explicitly compose the dynamics MDP with the monitor, with the resulting MDP having at most $|S \times S_\varphi |$ states. If one then includes the time step in the state, one could perform the soft-Bellman Backup directly on this automaton. In this composed automaton each (action, state) pair would need to be recorded. Thus, one would expect $O(|S \times S_\varphi \times A|)$ space to be used. In practice, this explicit representation is much bigger than $\mathcal {B}$ due to the BDDs ability to skip over time steps and automatically compress symmetries.

### Constructing $\mathcal {B}$

One of the biggest benefits of the BDD representation of a Boolean function is the ability to build BDDs from a Boolean combinations of other BDDs. Namely, given two BDDs with *n* and *m* nodes respectively, it is well known that the conjunction or disjunction of the BDDs has at most $n\cdot m$ nodes. Thus, in practice, if the combined BDD’s remain relatively small, Boolean combinations remain efficient to compute and one does not construct the full binary decision tree! Further, note that BDDs support function composition. Namely, given predicates $f(x_1, \ldots , x_n)$ and *n* predicates $g_i(y_1, \ldots , y_k)$ the function

38 $$\begin{aligned} f\bigg (g_1(y_1, \ldots , y_k), \ldots , g_n(y_1, \ldots , y_k)\bigg ) \end{aligned}$$

can be computed in time [16]:

39 $$\begin{aligned} O(n\cdot |B_f|^2\cdot \max _i{|B_{g_i}|}), \end{aligned}$$

where $B_f$ is the BDD for *f* and $B_{g_i}$ are the BDDs for $g_i$. Now, suppose $\hat{\delta }_1, \ldots \hat{\delta }_{\log (|S|)}$ are Boolean predicates such that:

40 $$\begin{aligned} \hat{\delta }(\mathbf {s}, \mathbf {a}, \mathbf {c}) = (\hat{\delta }_1(\mathbf {s}, \mathbf {a}, \mathbf {c}), \ldots , \hat{\delta }_{\log (|S|)}(\mathbf {s}, \mathbf {a}, \mathbf {c})). \end{aligned}$$

Theorem 1 and an argument similar to that for Corollary 1 imply then that constructing $\mathcal {B}$, using repeated composition, takes time bounded by a low degree polynomial in $|A\,\times \,S\,\times \,S_\varphi |$ and the horizon. Moreover, the space complexity before and after composition are bounded by Corollary 1.

### Evaluating Demonstrations

Next let us return to the question of how to evaluate the likelihood of a concrete demonstration in our compressed BDD. The key problem is that the BDD can only evaluate (binary) sequences of actions/coin flips, where as demonstrations are given as sequences of action/state pairs. That is, we need to algorithmically perform the following transformation.

41 $$\begin{aligned} s_0\mathbf {a}_0\mathbf {s}_1\ldots \mathbf {a}_{n}\mathbf {s}_{n+1} \mapsto \mathbf {a}_1\mathbf {c}_1\ldots \mathbf {a}_n \mathbf {c}_n \end{aligned}$$

Given the random bit model assumption, this transformation can be rewritten as a series of Boolean Satisfiability problems:

42 $$\begin{aligned} \exists ~ \mathbf {c}_i~.~\hat{\delta }(\mathbf {s}_i, \mathbf {a}_i, \mathbf {c}_i) = \mathbf {s}_{i+1} \end{aligned}$$

While potentially intimidating, in practice such problems are quite simple for modern SAT solvers, particularly if the number of coin flips used is small. Furthermore, many systems are translation invariant. In such systems, the results of a single query (42), can be reused on other queries. For example, in (29), $\mathbf {c} = \mathbf {0}$ always results in the agent moving to the right. Nevertheless, in general, if *q* coin flips are used, encoding all the demonstrations takes at most $O(|X|\cdot \tau \cdot 2^q)$, in the worst case.

### Run-Time Analysis

**Fig. 8.**
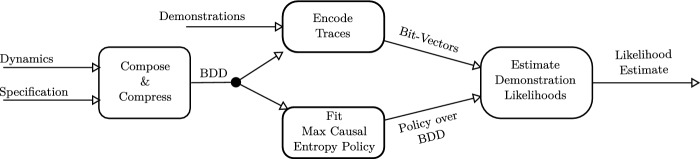
High level likelihood estimation procedure described in this paper.

We are finally ready to provide a run-time analysis for our new inference algorithm. The high-level likelihood estimation procedure is described in Fig. 8. First, the user specifies a dynamical system and a (multi-) set of demonstrations. Then, using a user-defined mechanism, a candidate task specification is selected. The system then creates a compressed representation of the composition of the dynamical system with the task specification. Then, in parallel, the maximum causal entropy policy is estimated and the demonstrations are themselves encoded as bit-vectors. Finally, the likelihood of generating the encoded demonstrations is computed.

There are three computational bottlenecks in the compressed scheme. First, given a candidate specification, $\varphi$, one needs to construct $\mathcal {B}$. As argued in Sect. 4.2, this takes time at most polynomial in the horizon, monitoring automata size, and MDP size (in the random-bit model). Second is the process of computing *Q* and *V* values by tuning the rationality coefficient to match a particular satisfaction probability. Just as with the naïve run-time (23), this process takes time linear in the size of $|\mathcal {B}|$ and logarithmic in the inverse tolerance $1/\epsilon$. Further, using Corollary 1, we know that $|\mathcal {B}|$ is at most linear in horizon and quasi-linear in the MDP size. Thus, the policy computation takes time polynomial in the MDP size and logarithmic in the inverse tolerance. Finally, as before, evaluating the likelihoods takes time linear in the number of demonstrations and the horizon. However, we now require an additional step of finding coin-flips which are consistent with the demonstrations. Thus, the compressed run-time is bounded by:

43 $$\begin{aligned} O\bigg ( \Big ( \underbrace{|X|}_{\#\text {Demos}} \cdot  \overbrace{\log \left( \epsilon ^{-1}\right) }^{\text {Feature Matching}}  \Big ) \cdot \text {POLY}\Big (  \overbrace{\tau }^{\text {Horizon}}  ,  \underbrace{|S|, |S_{|\varphi |}|, |A|}_{\text {Composed MDP size}}  ,  \overbrace{2^q}^{\#\text {Coin Flip Outcomes}}  \Big )\bigg ) \end{aligned}$$

#### Remark 8

In practice, this analysis is fairly conservative since BDD composition is often fast, the bound given by Corollary 1 is loose, and the SAT queries under-consideration are often trivial.

## Additional Model Refinements

### Conditioning on Valid Actions

So far, we have assumed that the number of actions is a power of 2. Functionally, this assumption makes it so each assignment to the action decision bits corresponds to a valid action. Of course, general MDPs have non-power of 2 action sets, and so it behooves us to adapt our method for such settings. The simplest way to do so is to use a 3-terminal Binary Decision Diagram. In particular, while each decision is still Boolean, there has now three possible types of leaves, 0, 1, and $\bot$. In the adapted algorithm, edges leading to $\bot$ are simply ignored, as they semantically correspond to invalid assignments to action or coin flip bits. A similar analysis can be done using these three valued decision diagrams, and as with BDDs, there exist efficient implementations of multi-terminal BDDs.

#### Remark 9

This generalization also opens up the possibility of state dependent action sets, where *A* is now the union of all possible actions, e.g, disable the action for moving to the right when the agent is on the right edge of the grid.

### Choice of Binary Co-Domain

One might wonder how sensitive this formulation is to the choice of $R(\xi ) = \theta \cdot \varphi (\xi )$. In particular, how does changing the co-domain of $\varphi$ from $\{0, 1\}$ to any other real values, i.e.,

$$\begin{aligned} \varphi ': (A \times S)^\tau \rightarrow \{a, b\}, \end{aligned}$$

change the likelihood estimates in our maximum causal entropy model. We briefly remark that, subject to some mild technical assumptions, almost any two real values could be used for $\varphi$’s co-domain. Namely, observe that unless both *a* and *b* are zero, the expected satisfaction probability, *p*, is in one-to-one correspondence with the expected value of $\varphi '$, i.e.,

$$ \mathop {\mathbb {E}}[\varphi '] = a\cdot p + b\cdot (1 - p). $$

Thus, if a policy is feature matching for $\varphi$, it must be feature matching for $\varphi '$ (and vice-versa). Therefore, the space of consistent policies is invariant under such transformations. Finally, because the space of policies is unchanged, the maximum causal entropy policies must remain unchanged. In practice, we prefer the use of $\{0, 1\}$ as the co-domain for $\varphi$ since it often simplifies many calculations.

### Variable Episode Lengths (with Discounting)

As earlier promised, we shall now discuss how to extend our model to include variable length episodes. For simplicity, we shall limit our discussion to the setting where at each time step, the probability that the episode will end is $\gamma \in (0, 1]$. As we previously discussed, this can be modeled by introducing a sink state, $\$$, representing the end of an episode (4). In the random bit model, this simply adds a few additional environment coin flips, corresponding to the environments new transitions to the sink state.

#### Remark 10

Note that when unrolled, once the end of episode transition happens, all decisions are assumed inconsequential w.r.t $\varphi$. Thus, all subsequent decisions will be compressed by in the BDD, $\mathcal {B}$.

Finally, observe that the probability that the episode ending increases exponentially, implying that the planning horizon need not be too big, i.e., the probability that the episode has not ended by timestep, $\tau \in \mathbb {N}$, is: $(1-\gamma )^{\tau }.$ Thus, letting $\tau = \lceil \ln (\nicefrac {\epsilon }{1 - \gamma }) \rceil$ ensures that with probability at least $1 - \epsilon$ the episode has ended.

## Experiment

Below we report empirical results that provide evidence that our proposed technique is robust to demonstration errors and that the produced BDDs are smaller than a naïve dynamic programming scheme. To this end, we created a reference implementation [29] in Python. BDD and SAT solving capabilities are provided via dd [21] and pySAT [12] respectively. To encode the task specifications and the random-bit model MDP, we leveraged the py-aiger ecosystem [28] which includes libraries for modeling Markov Decision Processes and encoding Past Tense Temporal Logic as sequential circuits.

**Fig. 9.**
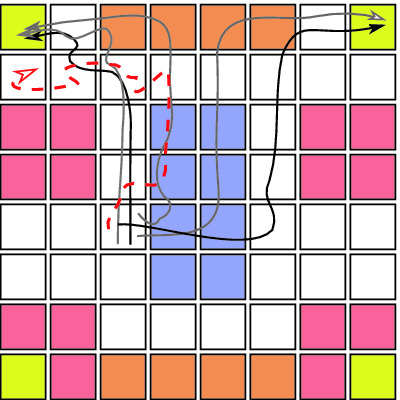
Example Gridworld (Color figure online)

**Problem:** Consider a gridworld where an agent can attempt to move up, down, left, or right; however, with probability 1/32, the agent slips and moves left. Further, suppose a demonstrator has provided the six unlabeled demonstrations shown in Fig. 9 for the task: “Within 10 time steps, touch a yellow (recharge) tile while avoiding red (lava) tiles. Additionally, if a blue (water) tile is stepped on, the agent must step on a brown (drying) tile before going to a yellow (recharge) tile.” All of the solid paths satisfy the task. The dotted path fails because the agent keeps slipping left and thus cannot dry off by $t=10$. Note that due to slipping, all the demonstrations that did not enter the water are sub-optimal.

**Table:** 

Spec	Policy size (#nodes)	ROBDD build time	Relative log likelihood (compared to true)
True	1	0.48s	0
$\varphi _1$ = Avoid lava	1797	1.5s	−22
$\varphi _2$ = Eventually Recharge	1628	1.2s	5
$\varphi _3$ = Don’t recharge while wet	850	1.6s	−10
	523	1.9s	4
	1913	1.5s	−2
	1842	2s	15
	577	1.6s	27

**Results:** For a small collection of specifications, we have computed the size of the BDD, the time it took to construct the BDD, and the *relative* log likelihoods of the demonstrations^4^,44where each maximum entropy policy was fit to match the corresponding specification’s empirical satisfaction probability. We remark that the computed BDDs are small compared to other straw-man approaches. For example, an explicit construction of the product of the monitor, dynamics, and the current time step would require space given by:

45 $$\begin{aligned} \tau \cdot |S|\cdot |A|\cdot |S_\varphi | = (10\cdot 8\cdot 8 \cdot 4) \cdot |S_\varphi | = 2560 \cdot |S_\varphi | \end{aligned}$$

The resulting BDDs are much smaller than (45) and the naïve unrolled decision tree. We note that the likelihoods appear to (qualitatively) match expectations. For example, **despite** an unlabeled negative example, the demonstrated task, $\varphi ^*$, is the most likely specification. Moreover, under the second most likely specification, which omits the avoid lava constraint, the sub-optimal traces that do not enter the water appear more attractive.

Finally, to emphasize the need for our causal extension, we compute the likelihoods of $\varphi ^*, \varphi _1, \varphi _2$ for our opening example (Fig. 1) using both our causal model and the prior non-causal model [30]. Concretely, we take $\tau = 15$, a slip probability of 1/32, and fix the expected satisfaction probability to 0.9. The trace shown in Fig. 1 acts as the sole (failed) demonstration for $\varphi ^*$. As desired, our causal extension assigned more than 3 times the relative likelihood to $\varphi ^*$ compared to $\varphi _1$, $\varphi _2$, and $true$. By contrast, the non-causal model assigns relative log likelihoods $(-2.83, -3.16, -3.17)$ for $(\varphi _1, \varphi _2, \varphi ^*)$. This implies that (i) $\varphi ^*$ is the least likely specification and (ii) each specification is less likely than $true$!

## Conclusion and Future Work

Motivated by the problem of learning specifications from demonstrations, we have adapted the principle of maximum causal entropy to provide a posterior probability to a candidate task specification given a multi-set of demonstrations. Further, to exploit the structure of task specifications, we proposed an algorithm that computes this likelihood by first encoding the unrolled Markov Decision Process as a reduced ordered binary decision diagram (BDD). As illustrated on a few toy examples, BDDs are often much smaller than the unrolled Markov Decision Process and thus could enable efficient computation of maximum causal entropy likelihoods, at least for well behaved dynamics and specifications.

Nevertheless, two major questions remain unaddressed by this work. First is the question of how to select which specifications to compute likelihoods for. For example, is there a way to systematically mutate a specification to make it more likely and/or is it possible to systematically reuse computations for previously evaluated specifications to propose new specifications.

Second is how to set prior probabilities. Although we have largely ignored this question, we view the problem of setting good prior probabilities as essential to avoid over fitting and/or making this technique require only one or two demonstrations. However, we note that prior probabilities can make inference arbitrarily more difficult since any structure useful for optimization imposed by our likelihood estimate can be overpowered.

Finally, additional future work includes extending the formalism to infinite horizon specifications, continuous dynamics, and characterizing the optimal set of teacher demonstrations.

## Footnotes

1 Formally, this is due to (a) softmax and average being monotonic (b) trajectory rewards only increasing with $\theta$, and (c) $\pi$ exponentially biasing towards high Q-values.

2 Mnemonic: $\mathcal {T}$ is a (typographically) slimmed down variant of $\mathbb {T}$.

3 See [31] for an explanation on systematically deriving such encodings.

4 The maximum entropy policy for $\varphi =\text {true}$ applies actions uniformly at random.
